# Thecodont tooth attachment and replacement in bolosaurid parareptiles

**DOI:** 10.7717/peerj.9168

**Published:** 2020-05-13

**Authors:** Adam J. Snyder, Aaron R.H. LeBlanc, Chen Jun, Joseph J. Bevitt, Robert R. Reisz

**Affiliations:** 1 Department of Biology, University of Toronto Mississauga, Mississauga, Ontario, Canada; 2 Department of Biological Sciences, University of Alberta, Edmonton, Alberta, Canada; 3 International Center of Future Science, Dinosaur Evolution Research Center, Jilin University, Changchun, China; 4 Lab for Evolution of Past Life and Environment in Northeast Asia, Jilin University, Ministry of Education, Changchun, China; 5 Australian Centre for Neutron Scattering, Sydney, Australia

**Keywords:** Thecodont, Bolosaurus, Beleby, Tooth attachment, Tooth replacement, CT, Histology, Dentary, Parareptile

## Abstract

Permian bolosaurid parareptiles are well-known for having complex tooth crowns and complete tooth rows in the jaws, in contrast to the comparatively simple teeth and frequent replacement gaps in all other Paleozoic amniotes. Analysis of the specialized dentition of the bolosaurid parareptiles *Bolosaurus* from North America and *Belebey* from Russia, utilizing a combination of histological and tomographic data, reveals unusual patterns of tooth development and replacement. The data confirm that bolosaurid teeth have thecodont implantation with deep roots, the oldest known such example among amniotes, and independently evolved among much younger archosauromorphs (including dinosaurs and crocodilians) and among synapsids (including mammals). High-resolution CT scans were able to detect the density boundary between the alveolar bone and the jawbone, as confirmed by histology, and revealed the location and size of developing replacement teeth in the pulp cavity of functional teeth. Evidence provided by the paratype dentary of *Belebey chengi* indicates that replacement teeth are present along the whole tooth row at slightly different stages of development, with the ontogenetically more developed teeth anteriorly, suggesting that tooth replacement was highly synchronized. CT data also show tooth replacement is directly related to the presence of lingual pits in the jaw, and that migration of tooth buds occurs initially close to these resorption pits to a position immediately below the functional tooth within its pulp cavity. The size and complex shape of the replacement teeth in the holotype of *Bolosaurus grandis* indicate that the replacement teeth can develop within the pulp cavity to an advanced stage while the previous generation remains functional for an extended time, reminiscent of the condition seen in other amniotes with occluding dentitions, including mammals.

## Introduction

Bolosaurid parareptiles occupy a unique position in amniote evolution. They were the first to develop a combination of a lower temporal fenestra and a massive coronoid process, probably for extensive oral processing (Reisz, 2006). The highly heterodont marginal dentition is characterized by a reduction in the number of teeth from the ancestral parareptile condition (e.g., two premaxillary, 10 maxillary, and 12 dentary teeth in the most complete specimen of *Belebey vegrandis,* Reisz et al., 2007), and the presence of extensive tooth-on-tooth wear, with the most massive cheek teeth located posteriorly along the tooth row. First described from Texas specimens, the three bolosaurid genera include two species of *Bolosaurus*, the smaller *Bo. striatus* (Cope, 1878) and more massive *Bo. grandis* (Reisz, Barkas & Scott, 2002) from deposits in the Southern United States. *Belebey* consists of three species, including two Russian forms. *Be. maximi* (Tverdokhlebova 1987) and *Be. vegrandis* (Ivakhnenko, 1973; Reisz et al., 2007), and the Chinese *Be. chengi* (Müller, Li & Reisz, 2008). Finally, the nearly complete *Eudibamus cursoris* (Berman, 2000) of Germany represents the only other known taxon within the group.

While *Bolosaurus* is mainly known from fragmentary cranial materials, and *Belebey* is known from complete cranial material, only *Eudibamus* is known from a complete articulated skeleton, which indicates that at least this bolosaurid was capable of bipedal locomotion, with a parasagittal gait (Berman, 2000). Despite their unusual dentition and gait, bolosaurids have attracted relatively little attention compared to other Palaeozoic tetrapods. Perhaps their most unusual characteristic is the apparent lack of evidence for tooth replacement, with no large replacement gaps along the tooth row, as is usually found in other Paleozoic tetrapods (Gordon Edmund, Edmund & Museum, 1960). Occasionally as in the holotype, crowns may be damaged, but part of the crown and the roots of the teeth are still present. Uninterrupted marginal tooth rows in the fossil record of Paleozoic tetrapods are rare, usually associated with unusual attachment and replacement cycles, such as in captorhinids (Bolt & DeMar, 1983; Reisz, Haridy & Müller, 2016). Parareptiles typically exhibit polyphyodonty with nearly continual tooth replacement cycles, producing frequent gaps along the tooth row (Gordon Edmund, Edmund & Museum, 1960; Macdougall & Modesto, 2011).

The level of heterodonty in bolosaurids is also remarkable, with mesial incisiform teeth transitioning to large, transversely bulbous cheek teeth distally along the tooth row. Tooth cusps slope posteriorly and have a conical apex that is more obvious in the larger, posterior teeth. Their presence in the early to middle Permian of Laurasia also marks one of the earliest known occurrences of dental occlusion within the fossil record of reptiles (Reisz, 2006), and their lack of denticles on the palate mark a divergence from the typical amniote condition of the time (Modesto, Scott & Reisz, 2009; Reisz, Godfrey & Scott, 2009; Müller & Reisz, 2005). The large, semilunar occlusal surfaces in bolosaurid teeth are often heavily worn, and have been interpreted as evidence for oral processing as specializations for intensive, fibrous herbivory (Sues & Reisz, 1998). All members of the clade have the same general dental pattern, presenting an exciting opportunity to examine the influence of occlusal methods of oral processing on dental evolution in early amniotes.

Attachment refers to the specific connection between the tooth and the tooth-bearing element. The geometry of that attachment defines the mode of tooth implantation (LeBlanc et al., 2017). The two are not exclusive, but trends do appear in the fossil record. In many amniotes, teeth are attached through ankylosis, or direct ossification. The tooth joins to the surrounding bone and is typically associated with acrodont (attached to the crest of the jaw) or pleurodont (attached to the labial wall of the jaw) implantation. By comparison, more derived, specialized thecodont implantations, are characterized by the formation of deep sockets. Thecodont teeth are also frequently attached via a soft tissue connection called a gomphosis. Ligament fibers suspend the tooth in the socket, reducing stress from mastication on the rest of the skull. These codevelop over multiple instances in the fossil record across various groups, including crocodilians, dinosaurs, and stem and crown mammals (LeBlanc et al., 2017; LeBlanc et al., 2018). Many other taxa display a mosaic of other attachment and implantation styles (Bertin et al., 2018).

Within bolosaurids, their unique dentition has been described as an ankylotic attachment, with a lack of plicidentine that is otherwise more typical of early Permian parareptile teeth (MacDougall, LeBlanc & Reisz, 2014). These descriptions were based on sections of a single species, *Bo. striatus*, from the Early Permian of Texas and represented a preliminary description of tooth attachment and implantation for bolosaurids. In order to further characterize tooth attachment, implantation, and replacement in bolosaurids, we examine here in detail the dental anatomy, processes of odontogenesis and tooth replacement, and a reevaluation of tooth implantation. Using a combination of histological and computed tomography (CT) from X-ray and Neutron energy sources, this paper reconstructs the patterns of tooth development and replacement in *Belebey* and *Bolosaurus*. This combination has rarely been applied with such high resolution to a targeted examination of teeth, dental tissues, and the associated jawbone (LeBlanc et al., 2020). In addition, the two methods have not been used in conjunction before to examine early amniotes with occluding dentition, providing insight into the earliest adaptations for fibrous diets. This data is enhanced by a new dentary fragment from the fossiliferous cave deposits in the Dolese Brothers Limestone Quarry, near Richards Spur, Oklahoma. Based on the clade’s anatomy, we also discuss their adaptations to herbivory and compare them to trends seen in other herbivorous stem amniotes. While not the earliest amniotes, these early parareptiles were the first to exhibit a number of traits that are maintained by crown amniotes millions of years later.

## Materials & Methods

Specimens were scanned under micro-CT and then processed in ImageJ before being segmented using Avizo Lite registered to R. R. Reisz. Neutron microtomography data were obtained for two dentaries of *Bolosaurus grandis*, OMNH 52311, ROMVP 83327; and a maxilla OMNH 15104, all from the Dolese Quarry, using the DINGO thermal-neutron radiography/tomography/imaging facility (Garbe et al., 2015) located at the 20 MW Open- Pool Australian Lightwater (OPAL) reactor housed at the Australian Nuclear Science and Technology Organisation (ANSTO), Lucas Heights, New South Wales, Australia. Data acquisition and reconstruction of the tomographic data were achieved using the method employed by Mays, Bevitt & Stilwell (2017). The two dentaries were scanned and reconstructed with a voxel size of 16.0 × 16.0 × 16.0 µm, and the maxilla with a voxel size of 45.4 × 45.4 × 45.4 µm; two dentaries of *Belebey chengi* IVPP V 12007, IVPP V 15907 from the Dashankou locality, China were X-ray scanned at IVPP, Beijing, with a voxel size of 29.8 × 29.8 × 29.8 µm.

The raw scan data is available at http://morphobank.org/permalink/?P3609.

To confirm tissue boundaries within bone seen in the CT scans, ROMVP 83327 was thin sectioned. This involved embedding the fossil in Castolite EP4101UV polyester resin and then curing for 24-hours. A Metcut-5 low speed saw equipped with a 5” diamond wafer blade cut a frontal plane slice of the anterior-most tooth and again along the sagittal plane. These were then mounted onto glass slides and cut using a Metcut-10 Geo low-speed diamond wafer blade. Specimens were ground using a grinding cup on the previous machine to 300 µm before being ground by hand using an abrasive block. Specimens were examined and photographed using a Leica MDG41 microscope with a crossed-polarizing filter and a combination of coaxial and reflected lights.

## Results

Dental tissues are difficult to characterize without destructive histological sampling, which is integral to understanding the processes of tooth attachment and development. Here we provide a brief description of the major dental tissues observed in most other amniotes and then compare these to our observations in our bolosaurid sample. The enamel acts as a veneer on the surface of the exposed tooth to help protect the inner dentine from wear. Reptilian enamel is typically very thin compared to that of mammals. In mammals, it often exceeds 1 mm in thickness, but in most reptiles, it is usually between 50–200 µm (Sander et al., 2000; Hwang, 2011). This is usually size-dependent as well; larger reptiles can have much thicker enamel (usually still not exceeding 1 mm), but in rare cases smaller reptiles (≤1 meter in body length) develop unusually thick enamel, exceeding 500 µm (Jones et al., 2018; LeBlanc et al., 2020). The dentine forms the bulk of the tooth, providing the structure to which the enamel binds and extending from the crown to the sites of attachment. Dentine is a tubular, avascular tissue in most amniotes. The tissue coating the dentine along the root surface is termed cementum: a thin mineralization around the roots with a cellular, mineral, and organic matrix composition similar to bone, but without the macroscopic nervous or venous systems (Bertin et al., 2018). Sometimes referred to as ‘bone of attachment’ to lump nearby connective tissues together, alveolar bone serves as both the primary support for the tooth and the field of growth for developing teeth (Smirina & Ananjeva, 2007). The alveolar bone forms the surrounding tooth socket to which the tooth is attached. The teeth may attach to the alveolar bone via a non-mineralized periodontal ligament (PDL), the anchoring ends of which form Sharpey’s fibres which penetrate through the alveolar bone and cementum (Budney, Caldwell & Albino, 2006; LeBlanc & Reisz, 2013; MacDougall, LeBlanc & Reisz, 2014; Bertin et al., 2018).

### Bolosaurus grandis

**Figure 1:**
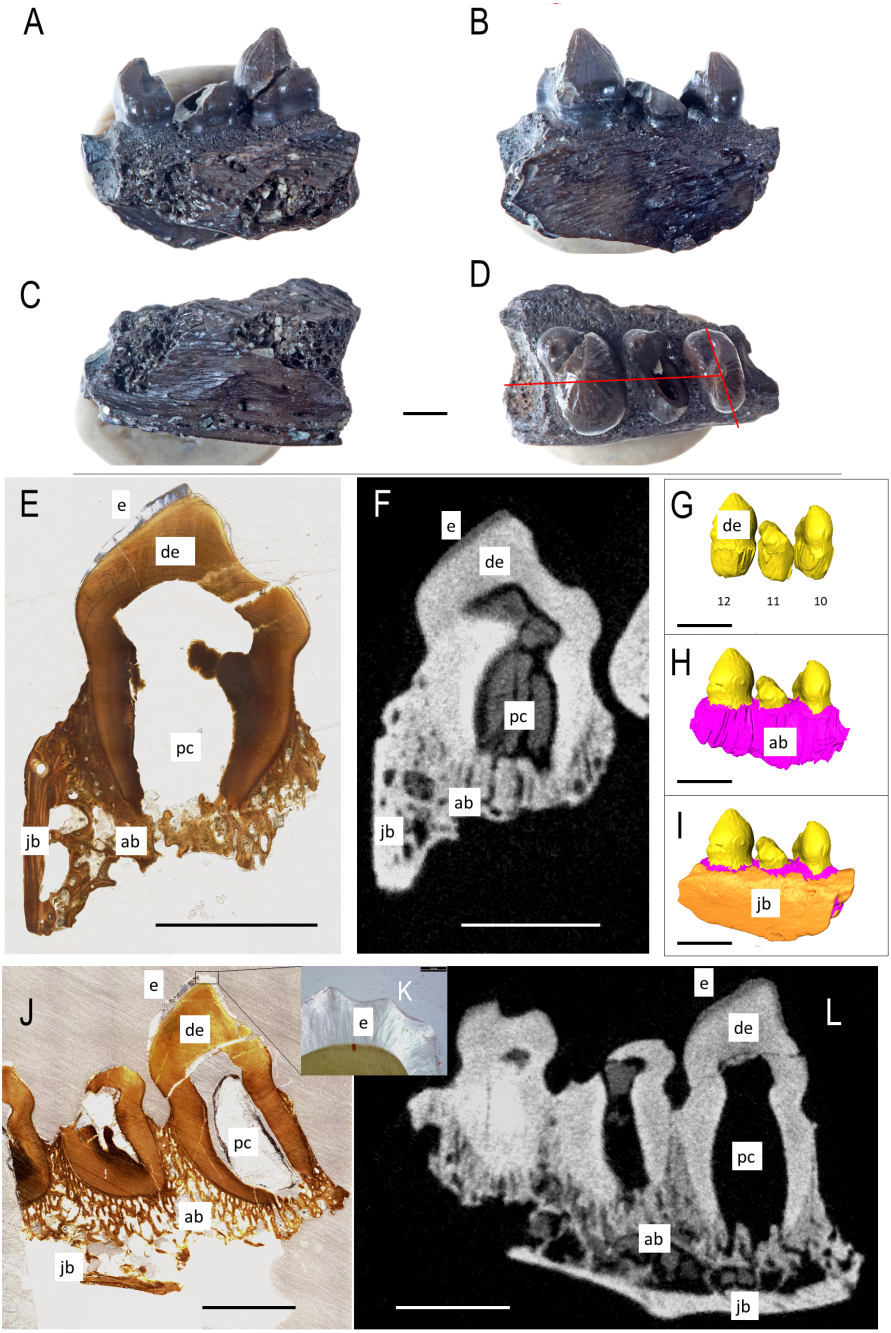
ROMVP 83327 *Bolosaurus grandis* right dentary fragment. (A) Lingual, (B) labial, (C) inferior, and (D) superior view with histological cuts marked in red. (E) Transverse histological view of inferred tooth locus 10. (F) Transverse digital thin section of inferred tooth locus. (G) Labial tooth segmentation. (H) Labial segmented profile with the addition of alveolar bone. (I) Complete labial dentary segmented profile. (J) Sagittal histological view of inferred tooth loci 11 and 12. (K) Close up of enamel structure. (L) Sagittal digital thin section of inferred tooth loci 11 and 12. ab, alveolar bone; de, dentin; e, enamel; jb, jawbone; pc, pulp cavity. Scale bars are 2.5 mm except 100 µm in K.

The lingual side of a right dentary fragment of *Bolosaurus grandis* [ROMVP 83327 (Fig. 1)] is damaged, exposing the trabecular structure of the alveolar bone. Three teeth are present and show enamel ridging that radiates from the apex in all directions, extending as far down the crown to the greatest horizontal extent of the tooth. This ridging is described extensively and is present on both *Bolosaurus grandis* and *Bo. striatus*, but is more pronounced in *B. grandis* (Reisz, 2006). Comparisons with the holotype of *Bo. grandis* reveals that the three teeth represent tooth loci 10, 11, and 12 in the dentary series. The first preserved tooth (tooth 10) has visible wear and past the apex of the cusp. The thickness of the enamel varied with a maximum extent of 205 µm related to the amount of visual occlusion and could be seen through histology and CT. The second tooth is broken on its mesial side, exposing the pulp cavity. This tooth did not have an extensive enamel covering, but this may have been the result of breakage. The final and most massive tooth is in the best condition, and also shows evidence of light wear. Neither method was able to reveal tissue that could confidently be associated with cementum in ROMVP 83327.

**Figure 2:**
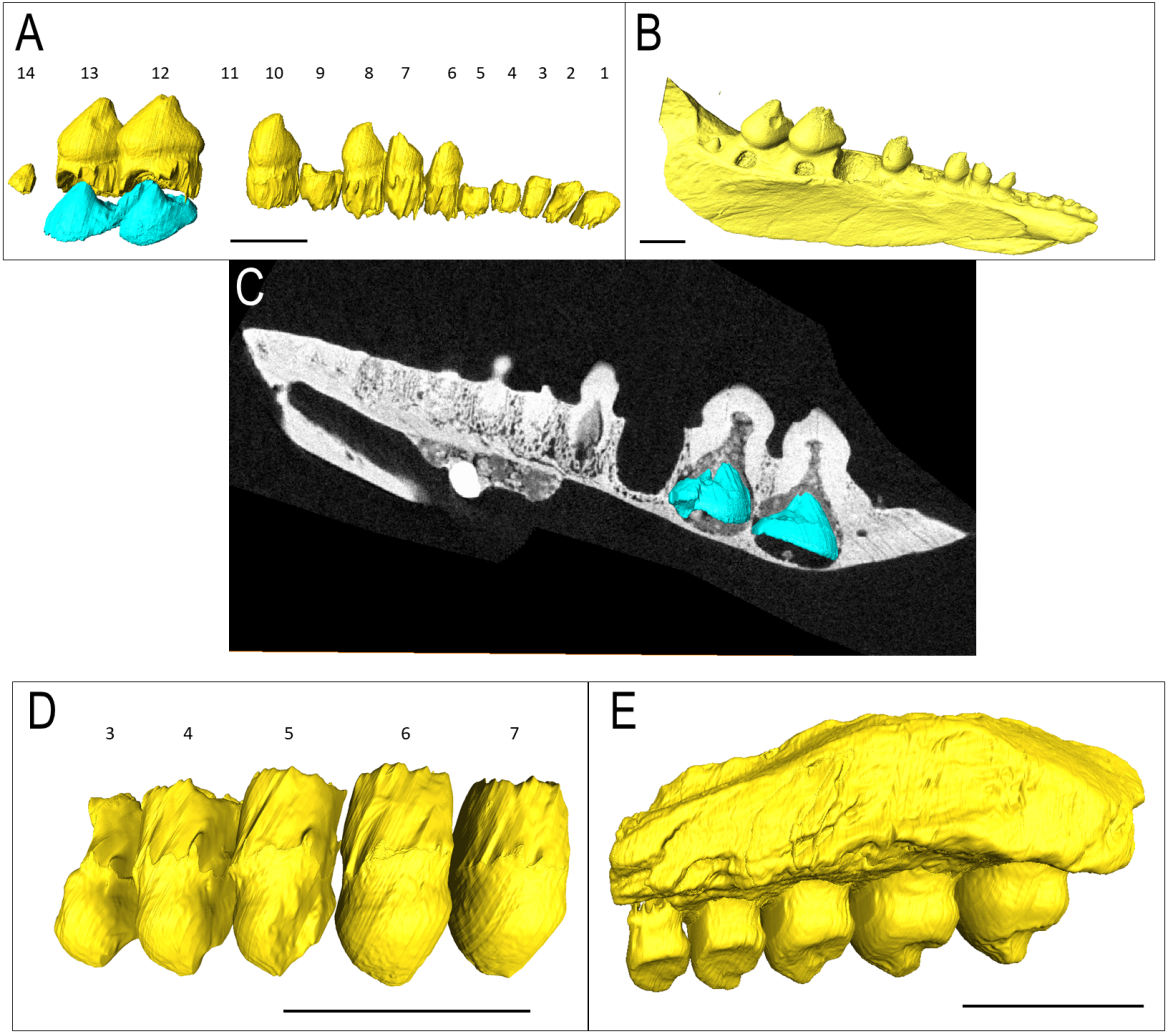
*Bolosaurus* CT scan data. *Bolosaurus grandis* holotype, OMNH 52311, dentary segmented in labial view (A) functional and developing teeth, and (B) complete isosurface. (C) Digital thin section of OMNH 52311 in labial view with segmented developing teeth. *Bolosaurus grandis*, OMNH 15104, maxilla segmented in labial view showing (D) inferred tooth loci 3–7 with no developing teeth and (E) complete isosurface. Scalebars 2.5 mm.

The isolated left dentary OMNH 52311 (Fig. 2) is the holotype for *Bolosaurus grandis* (Reisz, Barkas & Scott, 2002), and it possesses 14 tooth loci. Seven anterior teeth are broken near the bases of the crowns but still have well-preserved roots. The tooth crowns are ridged from their apex to their greatest lateral extent before the crown-root junction. As suggested in the original description, CT data show that the roots of the three anteriormost teeth are procumbent within the dentary, as seen in other bolosaurids (Reisz et al., 2007). The cheek teeth become more bulbous posteriorly along the dentary, but the most posterior tooth located at the base of the coronoid eminence in locus 14 is very small, unridged and peg-like. The digitally segmented roots of the teeth are proportional to the size of the crowns. Resorption pits are present on the dentary only at loci 12 and 13, which house developing successional teeth. No other tooth positions appear to have replacement teeth at any stage of development. The roots of the functional teeth at these loci show modest resorption. Locus 11 is a large, exposed resorption pit. The lack of any dentine, even at depth, suggests the tooth was shed and not broken. Locus 10 contains a small resorption pit directly below the pulp cavity; however, anterior positions are lacking these sites.

OMNH 15104 is a left maxillary posterior fragment, also included in the original description of *Bo. grandis* (Reisz, Barkas & Scott, 2002). It contains five complete teeth with no tooth gaps (Figs. 2D–2E). Rather than the typical posterolateral tooth cusps on the dentary, these teeth feature posteromedially directed cusps to support occlusion. The three anterior teeth show extensive lingual wear, appearing as a nearly flat surface compared to the concave shape present in the two posterior teeth. CT examination shows that a significant portion of the maxillary bone is missing, exposing the tooth and the alveolar bone.

### Belebey chengi

**Figure 3:**
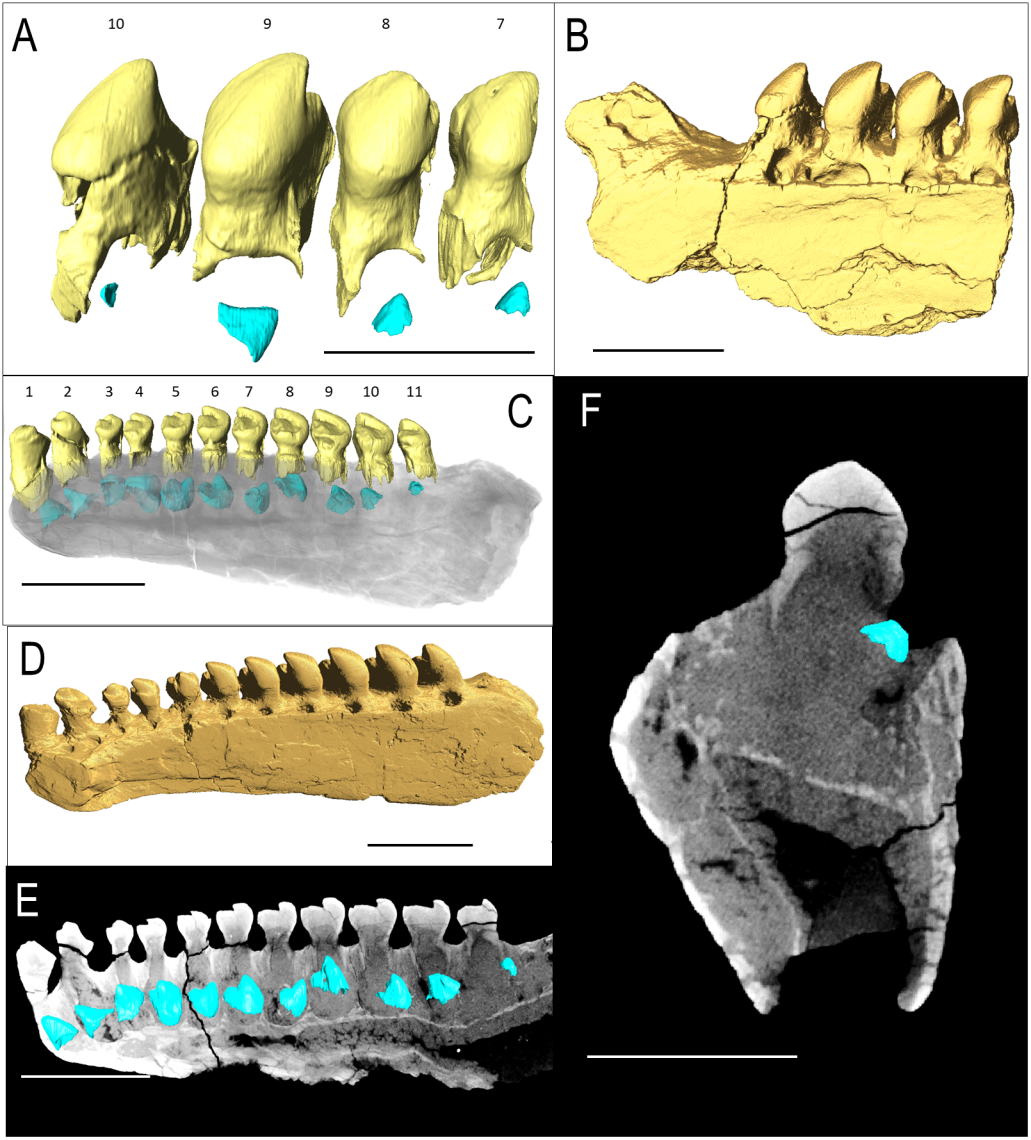
*Belebey* CT scan data. *Belebey chengis* specimen IVPP V 15907 segmented in lingual view to show (A) functional and developing teeth, and (B) complete isosurface. *Belebey chengis* specimen IVPP V 12007 in lingual view as a (C) transparent volume render with segmented functional and developing teeth, (D) complete isosurface and (E) Digital thin section segmented developing teeth. (F) Transverse digital thin section of IVPP V 12007 tooth locus 11. Scale bars 1 mm.

IVPP V 15907 is a posterior right dentary fragment of *Belebey chengi* (Müller, Li & Reisz, 2008: Figs. 3A–3B). An additional resorption pit appears behind the final tooth (Fig. 3B); however, there is no evidence of an extra tooth developing inside the dentary within this locus. Every locus contains a replacement tooth associated with a large resorption pit. While not as bulbous as *Bolosaurus grandis,* the teeth are expanded laterally along the tooth row and still have an occlusal surface at different stages of wear from the cusp to the lingual side along the mesial face.

**Figure 4:**
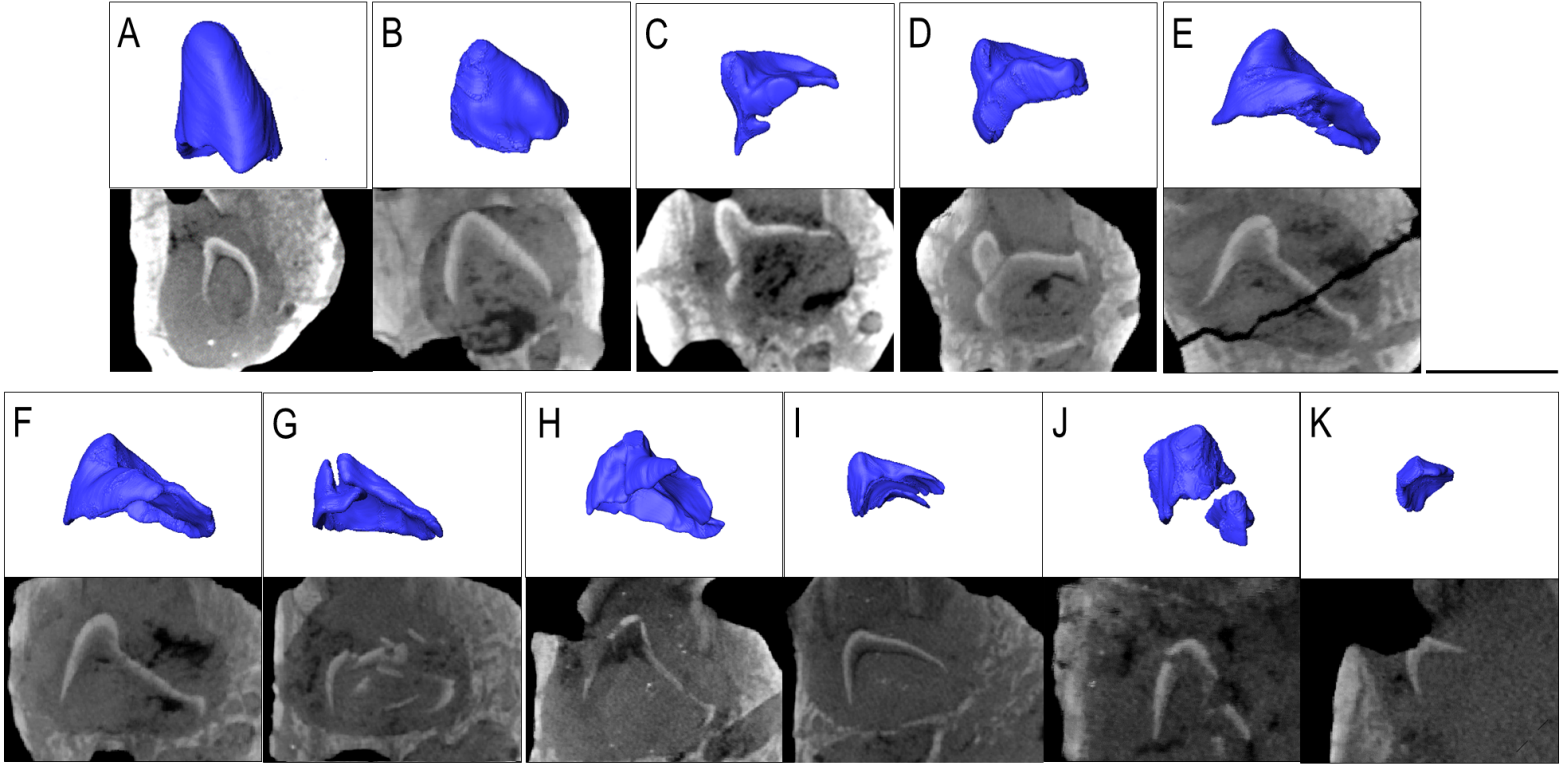
The replacement teeth of *Belebey* IVPP V 12007. Segmented profile above an apical digital thin section of developing teeth below (A) position 1, (B) position 2, (C) position 3 (D) position 4 (E) position 5 (F) position 6 (G) position 7 (H) position 8 (I) position 9 (J) position 10 and finally (K) position 11.

IVPP V 12007 is a right dentary of *B. chengi* (Müller, Li & Reisz, 2008: Figs. 3C–3F). This is a well-preserved specimen containing 11 teeth, and there are no gaps along the tooth row. Recurved cusps are heavily worn on the anterior five teeth, but posteriorly from locus six, the teeth show only slight wear on their crowns, and they form a pattern of successively increasing lateral size similar to that seen in *Bolosaurus*. In contrast to the condition seen in *Bolosaurus grandis*, the teeth are vertically oriented around the symphyseal area, rather than being inclined anteriorly. Resorption pits on the medial face of the bone are present at each tooth position, and they are relatively shallow and circular in outline. Heterodonty follows the same trend as in *Bolosaurus grandis,* but it is more gradual. Each tooth generally follows the same morphology as its predecessor along the jaw, with only slight modifications to their shape. CT scan data show that spacing between the developing teeth increases posteriorly along the dentary as the functional teeth occupy a greater surface area of the dentary bone (Fig. 4).

### Bolosaurid tooth attachment and implantation

The CT scan data show that the teeth have deep roots that are ankylosed to the jawbone (there is no space for a ligamentous connection between the teeth and the tooth sockets). Both the labial and lingual walls of the tooth roots are equally long, in contrast to the asymmetry between the labial and lingual sides commonly found in early amniotes (Gordon Edmund, Edmund & Museum, 1960; LeBlanc & Reisz, 2015). All the functional teeth have roots that extend far into the jaw but do not come into direct contact with the maxillary or dentary bone itself, remaining completely encased within the trabecular alveolar bone.

**Figure 5:**
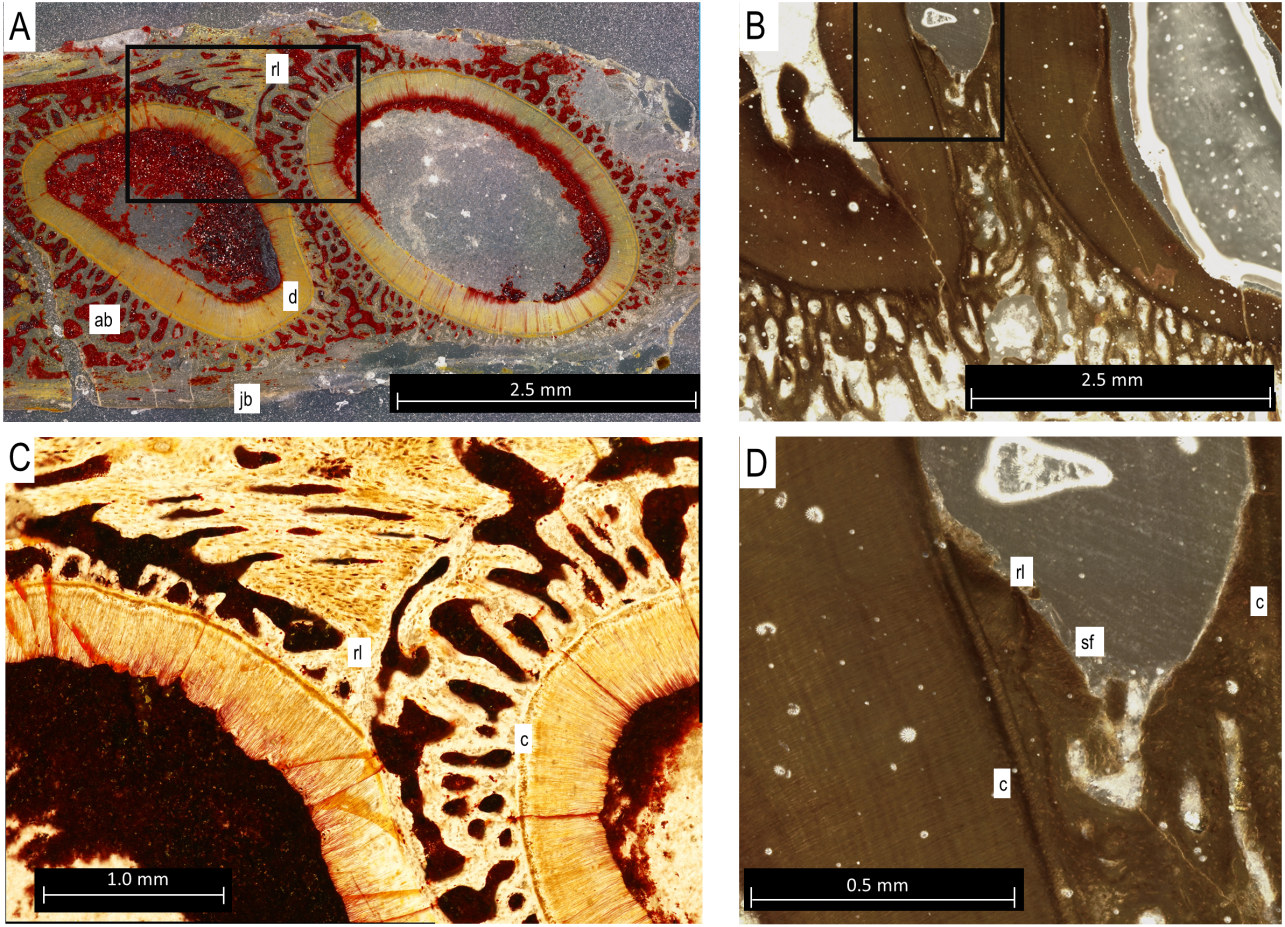
*Bolosaurus* thin sections. (A) Transverse section of StIPB-R 636 with a sharp contrast between alveolar and jawbone structure. (B) Sagittal cross section of ROMVP 83327. (C) Zoomed in section of region marked in 5A of specimen StlPB-R 636. (D) Zoomed in region of ROMVP 83327 in Fig. 5B. ab, alveolar bone; c, cementum; d, dentine; jb, jawbone; rl, reversal line; sf, Sharpey’s Fibers.

In histological sections, the alveolar bone surrounding each tooth is separated from the jawbone by a distinct reversal line (Figs. 5A and 5C), indicating that it is resorbed and redeposited with each tooth replacement event (LeBlanc & Reisz, 2013). The alveolar bone at each locus forms a circular layer of trabecular bone around the dentine root of the tooth. The alveolar bone from younger teeth crosses through the alveolar bone of older neighbouring tooth loci, even causing partial resorption of the dentine of older teeth in some cases (Figs. 5A and 5C). Under cross-polarized light, the alveolar bone is usually optically extinct, suggesting that it consists of a woven bone matrix (Fig. 5B). However, closer inspection reveals a network of parallel fibers within the alveolar bone that are interpreted as Sharpey’s Fibres extending towards the root surfaces (Fig. 5D). These partly mineralized fibers cross through the alveolar bone towards the tooth root surfaces. These fibers are small, but are visible in the alveolar bone of neighboring teeth (Fig. 5D). The dentine and surrounding alveolar bone do not make direct contact. The histological sections reveal a layer of acellular cementum around each tooth root (Figs. 5C and 5D). This comes into direct contact with the surrounding alveolar bone, supporting an ankylosis model of attachment. This is similar to tooth attachment in other early amniotes (LeBlanc & Reisz, 2015; LeBlanc et al., 2018).

### Enamel thickness and ornamentation

Contrast and resolution in the CT data were such that it was not possible to differentiate the cementum layer coating the tooth roots, but the thickest sections of enamel did appear in the CT. The combination of CT data and histological thin sections did permit a complete differentiation of dental and mandibular tissues of *Bo. grandis* (Fig. 1), including relatively thick layers of enamel. In the sagittal section, the enamel is non-uniform in thickness, showing occlusal wear on the labial face as well as ornamentation (Fig. 1K). The enamel-dentine junction (EDJ) is a smooth line throughout the crown, whereas the external surface of the overlying enamel is jagged, forming the external striations visible in many specimens. The differences in shape between the EDJ and the enamel surface indicate that the striations are formed by the enamel-producing cells and are not influenced by the underlying dentine whatsoever. At its thickest points, the enamel measures 205 µm thick measured perpendicular to the EDJ towards the outer surface of the enamel seen in Fig. 1K (Sander, 1999; Hwang, 2011). For comparative context, the enamel in bolosaurid teeth is much thicker than that of captorhinids, but is slightly thinner or comparable to the enamel of diadectids and many herbivorous dinosaurs. However, the teeth are much larger in diadectids and herbivorous dinosaurs.

### Bolosaurid tooth replacement

In the holotype of *Bolosaurus grandis,* there is a single locus, position 11, where the tooth is absent. The empty space is a rounded depression lined by alveolar bone (Fig. 2C). We interpret this as the penultimate stage of the tooth shedding process when the root of the functional tooth has been shed, but the replacement tooth has not yet fully erupted and fused in place. The replacement tooth likely fell out post-mortem before the specimen was buried.

The *Bolosaurus grandis* specimens all had deep roots surrounded by supporting tissues. Of the three specimens, only a single dentary, the holotype, had any unerupted teeth. The other two specimens, while still containing deep sockets, had no indications of developing teeth from the scans (Figs. 1G and 2D). In OMNH 52311, the developing teeth at the 12th and 13th tooth positions had already begun formation of the crown and are the two largest tooth crowns in the jaw (Figs. 2A–2C). The functional teeth show some resorption probably caused by their immediately growing replacement teeth in the same locus. As initially described for *Bo. striatus* (Watson 1954), the 14th tooth in *Bo. grandis* is significantly smaller and thinner with a different morphology from the neighboring teeth (Fig. 2A).

Contained within both of the *Belebey* specimens were complete sets of replacement teeth and resorption pits along the dentary (Figs. 3A, 3C, 3E and 4). Underneath each tooth locus, an unerupted replacement tooth is preserved at varying stages of dentine and enamel development. The degree of development of the replacement teeth suggests that they represent slightly different stages of tooth development along the jaw, with more developed replacement teeth located anteriorly. This suggests that new teeth formed in a sequential pattern from anterior to posterior in *Be. chengi*, unlike the alternating replacement waves that characterize most reptilian dentitions (Gordon Edmund, Edmund & Museum, 1960; Reisz, Haridy & Müller, 2016). The thickness of their enamel also gives an indication of their age, indicating that the more posterior replacement teeth along the dentary were less developed than those in the front of the dentary. Compared to both the anterior-most replacement tooth as well as the functional tooth in the same locus, the posterior-most developing tooth is the smallest and the youngest in the series in both specimens of *Be. chengi*.

The prevalence of replacement teeth in the complete dentary of *Belebey* (IVPP V 12007) also allows us to reconstruct the dynamics of tooth replacement in bolosaurids (Fig. 4). The youngest replacement tooth, at locus 11, is a thin cap of dentine and enamel positioned and angled at the opening of the resorption pit (Fig. 4K). This suggests that new teeth first formed near the alveolar margins of the jawbones, indicating that the tooth producing organ, the dental lamina, was situated on or near the surface of the alveolar bone, lingual to the tooth row. The replacement teeth then stimulated resorption of the alveolar bone and dental tissues along the lingual surface of functional teeth to create a resorption pit and eventually a foramina. Given the small size of the earliest-staged replacement teeth and the abundance of developing teeth within the pulp cavities of the functional teeth, (Fig. 4), this would suggest that new teeth spent significant proportions of their formative time nested deep within the jaws at a subdental position. This is unlike the condition in many other early amniotes (Gordon Edmund, Edmund & Museum, 1960). The developing teeth quickly migrated to a subdental position where they continued to develop and resorb the bases of the functional teeth, before eventually erupting and causing the functional tooth to be shed from below.

## Discussion

### Bolosaurid teeth were anchored via a thecodont ankylosis

This study utilized a combination of CT scanning and histology, applying both techniques to the same specimen to understand the limits of each. Thin sections clearly still revealed the microanatomical details of the teeth bones that were too fine even for CT scans (Fig. 5). However, for the distribution of major tissues, such as alveolar bone, dermal jawbone, enamel, and dentine, the CT data were more useful. As seen in Figs. 1E and 1F, the presence and even the thickness of the enamel on the cusps can adequately be determined. The thickness of the enamel could not be used reliably to determine the age of teeth due to differences in occlusional wear throughout the heterodont dentition (Reisz, 2006). The only expected tissues that were not found in CT were the cementum and the Sharpey’s fibers within the alveolar bone, which were visible in histological section (Figs. 5C and 5D). Utilizing CT is completely non-destructive and allows for unlimited views in all three axes. When a 3D scan is uploaded, a virtual copy is available for anyone, who can then manipulate or view the data; however they wish, in perpetuity. However, for detailed histological descriptions, incorporating both thin section and CT data are essential for highlighting a larger range of structures and tissue types.

Tooth implantation in bolosaurids is most similar to thecodonty (sensu LeBlanc et al., 2017; Bertin et al., 2018). The roots extend deep into the socket, surpassing the height of the crown (see Fig. 2). However, bolosaurids differ from many other thecodont taxa in the lack of unmineralized periodontal ligaments around the functional teeth. Once fully erupted, bolosaurid teeth rapidly fused to the jaw, mineralizing all of the surrounding attachment tissues (Fig. 5). Bolosaurids share the same implantation and attachment features with early Permian diadectids (LeBlanc & Reisz, 2013). Many other middle-to-late Permian amniotes, particularly therapsids, show similar thecodont implantation to bolosaurids, but the surrounding soft tissues do not mineralize, leaving a periodontal ligament space between the tooth root and the alveolar bone (Rybczynski & Reisz, 2001; LeBlanc et al., 2018; Whitney & Sidor, 2020). In bolosaurids, the teeth are firmly attached to the jawbone through the presence of alveolar bone. In CT, each socket appears to blend into the next with no distinct jawbone boundary between the walls continuously; however, in histological section, reversal lines exhibit a well-defined socket. The roots in bolosaurids are clearly defined within the jawbone; however, the alveolar bone shows previous dentine resorption of even other tooth loci, as divided by reversal lines (Fig. 5A). This permits a sequential interpretation of tooth replacement as the younger material crosscuts the older attachment tissues. This appears to be universal to all early amniotes, appearing in synapsids, captorhinids and diadectids (LeBlanc & Reisz, 2015).

This deep implantation of the teeth is unique among early parareptiles, as is the novelty of dental occlusion (Reisz, 2006; MacDougall, LeBlanc & Reisz, 2014). The wear created by occlusion may have been mitigated by the tooth implantation. Deep implantation of teeth is relatively rare among Permo-Carboniferous tetrapods. One comparable taxon are the diadectids. Dentitions in both diadectids and bolosaurids show heterodonty, thecodont implantation, as well as evidence of occlusal wear (LeBlanc & Reisz, 2013; MacDougall, LeBlanc & Reisz, 2014). The molariform cheek teeth of *Diadectes* are mediolaterally expanded compared to the more teardrop-shaped of bolosaurids, but like bolosaurids, they also frequently possess numerous replacement pits along the length of their jaws, suggesting a similar origin of tooth buds in both taxa.

Heterodonty, as well as the novel methods of tooth implantation and replacement, make the dentition of bolosaurids unique among early Permian parareptiles. The attachment tissues themselves match those of other early stem and crown amniotes (LeBlanc & Reisz, 2013; LeBlanc & Reisz, 2015; LeBlanc et al., 2018). The appearance of Sharpey’s fibers perforating the alveolar bone indicates that at some point in replacement, the teeth remain suspended by periodontal ligaments. During the development of a tooth underneath the functional tooth, the resorption required for growth removes hard calcified elements and replaces them with soft tissue. This soft tissue well is open to the surface through resorption pits until the functional tooth is shed, exposing the much greater extent of the tissue underneath. This large replacement pit supports the developing tooth, attached by soft tissues allowing for the movement into a functional position. Eventually, with tooth maturation, these soft elements undergo centripetal mineralization as the now harder alveolar bone grows into direct contact with the cementum and the tooth is fully ankylosed to the jaw. These ligamentous channels become surrounded by bone, preserved as Sharpey’s Fibres, existing as a few remnants in the resorbed material. Teeth are inferred to undergo direct ankylosis to the tissues resulting in firm anchoring to the jaw. This is in contrast to gomphosis, the primary method of attachment for later thecodonts such as mammals. It has been inferred that these soft-tissue attachments for teeth evolved in conjunction with occlusion to limit mechanical stress (Gaengler & Metzier, 1992). Bolosaurids require a separate, possibly different explanation as intensive dental occlusion wears down the teeth (Reisz, 2006), but no analogous supporting periodontal ligamentous tissues are present in functional tooth attachment.

### Mode and timing of tooth replacement in Bolosauridae minimized gaps in their occluding dentition

Thecodont taxa display two different types of tooth replacement. The first occurs in modern mammals and crocodilians, wherein a replacement tooth develops from a dental lamina that is dissociated from the oral epithelium and is buried deep within the jaw, almost underneath the functional teeth (Gordon Edmund, Edmund & Museum, 1960). The second replacement type is found in most other thecodont amniotes, where the dental lamina is still located along the lingual margins of the jaws, an ancestral feature for amniotes (Gordon Edmund, Edmund & Museum, 1960; LeBlanc et al., 2017; Leblanc, Mohr & Caldwell, 2019; Whitney & Sidor, 2020). Because new teeth still form far away from the pulp cavity of the preceding functional teeth, they first form resorption pits along the lingual surfaces of the teeth that are visible externally in fossils and skeletons. These resorption pits are the entry points for replacement tooth buds, which then mature beneath the functional tooth. This is the condition we observed in bolosaurids. However, despite the prevalence of replacement teeth in our bolosaurid sample, vacant tooth positions are rare compared to other polyphyodont amniotes (Gordon Edmund, Edmund & Museum, 1960).

One specimen of *Belebey* (IVPP V 15907) shows replacement pits along the lingual side of every single tooth locus, but no shed teeth (Figs. 3C–3E). The least developed replacement teeth in IVPP V 15907 are close to the opening of the small replacement pit (Figs. 3F and 4K). This is strong evidence that the dental lamina existed along the lingual margin of the tooth row, periodically initiating a new tooth that quickly formed a replacement pit along the lingual surface of the functional tooth root and alveolar bone. The developing teeth would drop to a subdental position and continue to develop for some time before causing the functional teeth to be shed. Tooth shedding may have been a relatively quick process in the tooth replacement cycle, given that we only observed one empty tooth locus in our bolosaurid sample (Figs. 2A–2C). The prevalence of replacement teeth deep in the jaws in bolosaurids also suggests that replacement teeth spent a great deal of time developing in these subdental crypts before finally erupting and replacing a functional tooth. This mode of tooth replacement, where teeth rapidly descended into the jaws and spent significant time developing underneath the functional tooth row, would have extended the life of functional teeth, allowing them to remain in the mouth for a more extended amount of time without compromising precise occlusion.

The presence of replacement teeth in *Belebey* (IVPP 15104) and *Bolosaurus* (OMNH 52311) revealed an unusual tooth replacement pattern for bolosaurids. The presence of regularly arranged tooth gaps in fossil reptiles represent a period in the replacement cycle in which the functional tooth has been shed, but the replacement tooth had not fully erupted at the time of death (Gordon Edmund, Edmund & Museum, 1960). At first glance, the dentary of *Belebey* (IVPP 15104) shows replacement teeth at nearly the same stage of development along the jaw (Fig. 3). It is possible that bolosaurids might have replaced all tooth positions rapidly and simultaneously, but this is an exceptionally rare occurrence in tetrapods (Bolt & DeMar, 1983) and would be extremely detrimental to an animal that relied on dental occlusion. As opposed to showing intermittent or large gaps in the tooth row, bolosaurids usually possess a gapless dentition or only a single shed tooth position. Moreover, closer inspection of the replacement teeth in *Belebey* (IVPP 15104) and *Bolosaurus* (OMNH 52311) reveal that, unlike most other polyphyodont amniotes, bolosaurids underwent a single replacement wave at a time, passing from the front of the jaw to the back (Figs. 2–4). Each replacement tooth in *Belebey* is incrementally more developed from the front of the jaw to the back. This single-wave model also explains why in OMNH 52311, there is no evidence of resorption pits in the anterior 10 teeth, tooth 11 has been shed, and posterior tooth positions 12 and 13 were still undergoing replacement. The replacement wave in this specimen had already passed through the first 10 teeth, while tooth 11 had recently been shed. Meanwhile, positions 12 and 13 have very large and well-developed replacement teeth and were likely nearing the tooth shedding phase (Fig. 2). As opposed to periodically replacing the entire dentition and being unable to feed for a short time, we hypothesize that bolosaurids instead replaced a single tooth position at a time, which would minimize the impact of tooth replacement on the occlusal relationships of the teeth. This proposed method assumes a reasonably equal rate of tooth development across the jaw, but the much smaller, less complex anteriormost teeth would reach eruption before the much larger posterior teeth even reached maturation, due to the variation in size in the heterodont dentition (Reisz et al., 2007).

### Bolosaurid enamel is unusually thick for a small reptile

The enamel of bolosaurid teeth is thick and well ornamented (Fig. 1K). The crests at the crown apex are the thickest regions of the enamel, attaining a maximum thickness of approximately 200 µm. These ridges are clearly produced during the later stages of amelogenesis, and not during the initial phases of tooth development. We can infer this because the underlying dentine, the first hard tissue to be produced, does not conform to the shape of the outer surface of the enamel. The crowns must initially have a smooth outer shape that is later furnished with thicker crests of enamel. Whereas bolosaurid enamel is not particularly thick in absolute standards, it is extremely thick relative to the small sizes of its teeth. Many large, herbivorous dinosaurs, for example, have only slightly thicker, or even thinner enamel than these small parareptiles (Hwang, 2011). Thickened and wrinkled enamel is also unusual in small-bodied animals and may be an adaptation for occlusion and more powerful bites (Sander, 1999).

## Conclusion

Bolosaurids truly are a unique group of early reptiles with an unusual patchwork of dental characteristics. They exhibit not only strong heterodonty and extensive tooth-on-tooth wear but also have deeply implanted, thecodont teeth that are ankylosed to the jaw. Bolosaurids apparently maintained tooth-on-tooth wear over prolonged periods of time through specialized tooth replacement cycles. Although it appears that tooth development is nearly synchronous, our CT- and histology-based analyses show that the anterior teeth were replaced earlier than the larger posterior teeth in what appears to be a single tooth replacement wave, passing from the front of the jaw to the back. As in the stem amniote *Diadectes* (Gordon Edmund, Edmund & Museum, 1960), the thecodont condition seen in bolosaurids and the lack of gaps in the tooth row appear to be related to the need to maintain the occlusal relationships of the upper and lower teeth. As in extant mammals and crocodilians, new teeth grew deep within the jaw beneath the functional tooth, but they apparently migrated into this position from a lingually located dental lamina, as in other non-crocodilian reptiles. During the eruption of the new replacement teeth, the teeth were supported by ligamentous soft tissue that completely mineralized to form a dental ankylosis, as in most other Paleozoic reptiles. These features, coupled with their thickened and ornamented enamel crowns, reveal that bolosaurids were well adapted for extensive oral processing, a feature that was extremely rare among early Permian amniotes.

## Institutional Abbreviations

ANSTO: Australian Nuclear Science and Technology Organization
IVPP: Institute of Vertebrate Paleontology and Paleoanthropology
JLU: Jilin University
OMNH: Oklahoma Museum of Natural History
ROM: Royal Ontario Museum
StIPB-R: Steinman Institute Division of Paleontology

## Additional Information and Declarations

### Data Availability

The following information was supplied regarding data availability: The raw scan data is available at https://morphobank.org/index.php/Projects/ProjectOverview/project_id/3609. - M683226–M683236: OMNH 52311, ROMVP 83327, and OMNH 15104 - M683237: IVPP V 15907 - M683238 : IVPP V 12007 OMNH: Oklahoma Museum of Natural History IVPP: Institute of Vertebrate Paleontology and Paleoanthropology ROM: Royal Ontario Museum
